# In Vivo Imaging of Click-Crosslinked Hydrogel Depots Following Intratympanic Injection

**DOI:** 10.3390/ma13143070

**Published:** 2020-07-09

**Authors:** Hyeon Jin Ju, Mina Park, Ji Hoon Park, Gi Ru Shin, Hak Soo Choi, Myung-Whan Suh, Moon Suk Kim

**Affiliations:** 1 Department of Molecular Science and Technology, Ajou University, Suwon 443-749, Korea; tssos@ajou.ac.kr (H.J.J.); jhp@ajou.ac.kr (J.H.P.); mirage1008@ajou.ac.kr (G.R.S.); 2 Department of Otorhinolaryngology-Head and Neck Surgery, Seoul Medical Center, Seoul 05505, Korea; pimpleboy@naver.com; 3 Gordon Center for Medical Imaging, Department of Radiology, Massachusetts General Hospital and Harvard Medical School, Boston, MA 02114, USA

**Keywords:** intratympanic injection, hyaluronic acid, click-crosslinking, near-infrared fluorescence, depot

## Abstract

In this study, we developed injectable intratympanic hyaluronic acid (HA) depots for the treatment of hearing loss. We prepared an injectable click-crosslinking formulation by modifying HA with tetrazine (HA-TET) and trans-cyclooctene (HA-TCO), which crosslinked to form an HA depot (Cx-HA). Preparation of the click-crosslinking HA formulation was facile, and Cx-HA depot formation was reproducible. Additionally, the Cx-HA hydrogel was significantly stiffer than HA hydrogel. To monitor the degradation pattern of hydrogels, we mixed a zwitterionic near-infrared (NIR) fluorophore (e.g., ZW800-1C) in the click-crosslinking HA formulation. Then, HA-TET and HA-TCO solutions containing ZW800-1C were loaded separately into the compartments of a dual-barrel syringe for intratympanic injection. The Cx-HA depots formed quickly, and an extended residence time in the tympanic cavity was confirmed by performing NIR fluorescence imaging. We have successfully prepared an injectable click-crosslinking HA formulation that has promise as an intratympanic drug depot.

## 1. Introduction

Partial or complete hearing loss can be caused by inner ear disease, aging, exposure to toxins, noise, or Meniere’s disease, which may result in sudden hearing loss [1]. Hearing loss is a common problem and a social issue because its impacts are not confined to hearing loss patients. At present, the standard treatment for hearing loss is oral steroid administration [2]. However, very little of the steroid reaches the sites associated with hearing loss, which can severely limit its therapeutic efficacy [3,4,5]. Oral steroid treatment thus requires the administration of large doses, and one third of hearing loss patients experience systemic side effects. Approximately one percent of patients experience serious complications, such as hip fractures, toxic hepatitis, and death [6].

The intratympanic injection of steroids in high doses has recently been investigated as a hearing loss treatment [7,8,9]. A steroid solution is injected into the tympanic cavity, or middle ear, through the tympanic membrane. The injected steroid solution must then travel from the tympanic cavity to the inner ear. However, the steroid solution injected into the tympanic cavity could be discharged within tens of minutes through the auditory canal. As a consequence, the therapeutic effect of the steroid on the inner ear may not be sufficient [10]. To treat hearing loss, formulations that maintain a high steroid concentration in the tympanic cavity for a sufficient amount of time must be developed.

Several research teams, including our group, have recently reported the results of injecting hydrogels that form depots in situ [11,12,13]. In this study, we investigated the direct intratympanic injection of hydrogels that could form depots in situ to achieve a high steroid concentration at the location responsible for hearing loss. 

Hydrogels that can form depots in situ are typically comprised of natural biomaterials, such as hyaluronic acid (HA), collagen, and fibrin, and various synthetic biomaterials [14,15,16,17,18]. HA is a common ingredient in formulations that have been approved by the Food and Drug Administration (FDA) for various medical applications. HA solutions can readily be prepared as injectable formulations. A hydrogel depot forms at the injection site, but the depot disappears rapidly under physiological conditions. The short residence times of HA depots at injection sites may thus necessitate multiple injections.

Our group and other research teams have developed systems to utilize the biorthogonal click-crosslinking Diels–Alder reaction between tetrazine (TET) and trans-cyclooctene (TCO), because the reaction proceeds rapidly under physiological conditions [19,20,21,22]. We modified HA with TET (HA-TET) and TCO (HA-TCO) to prepare injectable click-crosslinking HA formulations. When HA-TET and HA-TCO are mixed, they form a crosslinked HA (Cx-HA) depot. This technique has the advantages of facile handling and reproducibility, and the residence times of Cx-HA depots are comparatively long [23,24,25].

**Figure 1:**
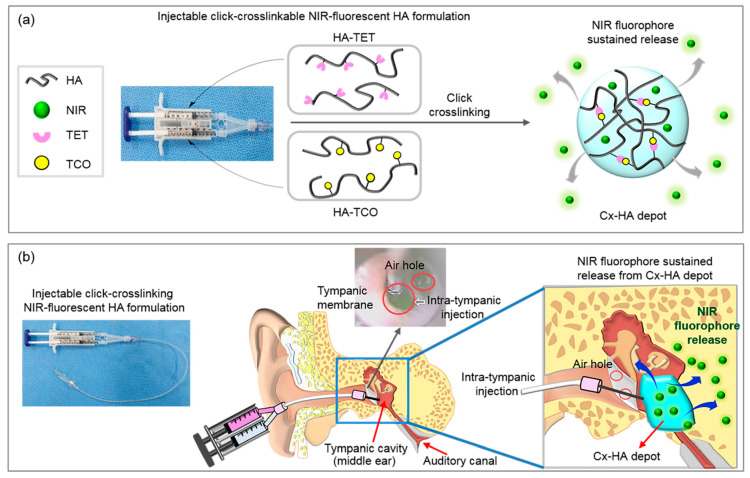
(**a**) Schematic illustration showing the injection of the click-crosslinking HA-TET and HA-TCO formulation and the sustained release of NIR fluorophores from a Cx-HA depot. (**b**) Intratympanic injection of the click-crosslinking NIR-fluorescent HA formulation and depot formation in the tympanic cavity. J.H.J., G.R.S., and M.S.K. created the illustrations using Adobe Photoshop 7.0 (San Jose, CA, USA).

Near-infrared (NIR) fluorescence imaging is a widely used and effective imaging technique [26], and ZW800-1C is a biocompatible NIR fluorophore that affords high sensitivity and resolution [26,27,28,29]. We thus chose ZW800-1C to study the efficacy of Cx-HA depots. To the best of our knowledge, this is the first report to formulate click-crosslinking HA with an NIR fluorophore for intratympanic injection (Figure 1).

We performed NIR fluorescence imaging after injecting the click-crosslinking NIR fluorescent HA formulation into the tympanic cavity of rats to investigate the formation and residence time of the Cx-HA depots. Our findings will facilitate the efficient development of click-crosslinking HA hydrogel formulations for drug delivery via intratympanic injection to address unmet needs for the treatment of hearing loss.

## 2. Materials and Methods 

### 2.1. Modification of HA with Tetrazine (HA-TET) and Trans-Cyclooctene (HA-TCO)

HA powder (100 mg; Humedix Inc. Anyang, Korea) was added to separate tubes along with deionized water (DW, 10 mL). 4-(4,6-Dimethoxy-1,3,5-triazin-2-yl)-4-methyl-morpholinium chloride (DMTMM) (70 mg, 0.24 mmol; Sigma, St. Louis, MO, USA) was added to each HA solution to activate the carboxyl groups in HA. After stirring for 30 min, tetrazine (3.63 mg, 0.01 mmol; Click Chemistry Tools, Scottsdale, AZ, USA) and TCO (2.63 mg, 0.01 mmol; Click Chemistry Tools) were added to each activated HA solution (10 mg/mL). The reaction mixtures were stirred for 24 h, then individually dialyzed for 72 h to remove unreacted TET and TCO. Following dialysis, the HA-TET and HA-TCO mixtures were lyophilized in a FD 8508 freeze-dryer (Ilshinlab, Daejeon, Korea).

### 2.2. Preparation of Cx-HA

Individual HA-TET and HA-TCO solutions (20 mg/mL) were prepared by dispersing the solid compounds in phosphate-buffered saline (PBS, pH 7.4). The solutions were loaded separately into the compartments of a dual-barrel syringe. The solutions in the dual-barrel syringe were injected simultaneously into a vial to provide Cx-HA for subsequent characterization.

### 2.3. Characterization of HA and Cx-HA

The rheological properties of HA and Cx-HA were evaluated using an MCR 102 rheometer (Anton Paar, Ostfildern, Germany). The measurement system was equipped with a temperature-controlled Peltier bottom plate and a 25.0 mm stainless steel parallel plate. A gap length of 0.3 mm was applied for all measurements, which were conducted at 25 °C. Measurements were performed over a 0 to 100 s time sweep at an oscillation frequency of 1 Hz and an amplitude (γ) of 2%. An oscillation frequency sweep from 0.1 to 10 Hz was also conducted (2% γ). Oscillation strain was measured from 0.1 to 1000% at an oscillation frequency of 1 Hz. The storage modulus (G′), loss modulus (G′′), phase angle (tan δ), and viscosity (η) were calculated using the software provided with the instrument.

### 2.4. Loading HA-TET and HA-TCO with the ZW800-1C NIR Fluorophore

The ZW800-1C NIR fluorophore was prepared using previously reported methods [26]. ZW800-1C (188.6 μg, 0.2 μmol) was dissolved in separate HA-TET and HA-TCO solutions (200 μL). The compartments of a dual-barrel syringe were loaded separately with the NIR-fluorescent HA-TET and NIR-fluorescent HA-TCO solutions for subcutaneous or intratympanic injection. The NIR fluorophore (377.2 μg, 0.4 μmol) was also dissolved in saline (400 μL) and loaded into a single-barrel syringe.

### 2.5. Subcutaneous Injection of the NIR-Fluorescent HA Formulation In Vivo

The protocols used in this study were approved by the Ajou University School of Medicine Institutional Animal Experiment Committee (Approval No. 2013-0070). Six-week-old male nude mice weighing 20–22 g each were used in accordance with the approved guidelines. The mice were anesthetized with zoletil and rompun (1:1) at a dose of 1.5 mL/kg and injected subcutaneously on their dorsal sides with either NIR-fluorescent HA-TET and NIR-fluorescent HA-TCO or the NIR fluorophore solution. In vivo optical and NIR imaging of the mice injected with either the NIR-fluorescent HA formulation or the NIR fluorophore solution was performed in real time on days 1, 2, 3, 4, 5, and 8. The fluorescence excitation wavelength was 730 nm, and emission was monitored in the range from 750 to 825 nm by passing the emitted light through a bandpass filter. A dichroic MgF2 fused silica filter was used for imaging with an exposure time of 3000 ms and a gain of ten. An NIR fluorescence image was captured at each time point using a FOBI imaging instrument (NeoScience, Suwon, Korea). The mice were sacrificed eight days post-injection, and the Cx-HA depots were excised from the injection sites to evaluate their in vivo persistence.

### 2.6. Intratympanic Injection of the NIR-Fluorescent HA Formulation In Vivo

All procedures in this study were performed after approval by the Institutional Animal Care and Use Committee (#0720142128). All Sprague Dawley rats (280–300 g, 6 weeks) were anesthetized with zoletil and xylazine, and their tympanic membranes were examined using an OPMI Pico surgical microscope (Zeiss, Oberkochen, Germany). A 10 cm long flexible Mini-Volume extension line (Insung Medical, Seoul, Korea) was connected to an Angiocath Plus 24 gauge needle (BD, Sandy, UT, USA) on one end. The other end of the line was connected to either a dual-barrel syringe loaded with NIR-fluorescent HA-TET and NIR-fluorescent HA-TCO or a single-barrel syringe loaded with the NIR fluorophore solution. Either the NIR-fluorescent HA formulation or the NIR fluorophore solution was injected through the intratympanic membrane into the tympanic cavity (middle ear). A syringe was used to create air vents in the anterior superior quadrants of the tympanic membranes. The posterior upper quadrants of the tympanic membranes were then injected with 30–60 μL of either the NIR-fluorescent HA formulation or the NIR fluorophore solution at a speed of approximately 4 μL/s. Injection was stopped when the tympanic cavity was completely filled or the NIR-fluorescent HA formulation or NIR fluorophore solution leaked through the air vent.

### 2.7. Measurement of the Auditory Brainstem Response (ABR)

The animals were anesthetized as described previously, and the ABR of each animal was measured after stimulating both the injected and non-injected ears to monitor changes in hearing. The ABR measurements were performed in a sound-proof chamber using a Smart EP system (IHS, Miami, USA). An active subdermal needle electrode was inserted at the vertex of the injected ear, and the reference electrode was inserted behind the same ear. The ground electrode was inserted in the opposite ear. The speaker was aligned with the external auditory canal, and the earphone tube was gently inserted into the ear canal. Click auditory stimuli were then delivered to the target ear. Hearing thresholds were determined by identifying the lowest levels at which auditory stimuli were recognized. The responses to III, V, and slow negative (SN10) waves were recorded as the sound pressure level (SPL) was reduced from 90 dB SPL in 5 dB increments. The ABR was measured twice for each ear, and the ABR threshold was recorded as the average of the two measured values. Two different researchers blindly evaluated the waveforms to compare the ABRs for the ears that received intratympanic injections and ears that were not injected.

### 2.8. Endoscopic Observation of Fluorescence in the Tympanic Membrane

Optical and NIR fluorescence images of the ears were acquired on days 1, 2, 3, 4, 5, and 8 following intratympanic injection of either the NIR-fluorescent HA formulation or the NIR fluorophore solution. Anatomic localization for optical imaging was performed using a conventional visible-light endoscope. Real-time fluorescence endoscopy was performed simultaneously to collect NIR fluorescence images of the NIR-fluorescent Cx-HA depots and the NIR fluorophore solution in the tympanic cavities. The fluorescence endoscope images were recorded and processed using an InTheSmart real-time fluorescence imaging system (Seoul, Korea). The excitation wavelength and power were 808 nm and 2.5–6.0 W, respectively, and emission was monitored at 825 nm.

## 3. Results

### 3.1. Preparation and Characterization of Injectable Click-Crosslinking HA Hydrogels

To ensure that Cx-HA depots formed in the tympanic cavities, the carboxylic groups in HA were first activated. They could then react with TET and TCO to yield HA-TET and HA-TCO, respectively. The characteristic HA-TET and HA-TCO peaks were observed in their ^1^H nuclear magnetic resonance (NMR) spectra (data not shown).

HA-TET and HA-TCO readily dissolved in PBS, and each compound remained in solution for a long period of time at room temperature prior to mixing. When the HA-TET and HA-TCO solutions were mixed in a vial, HA-TET and HA-TCO quickly click-crosslinked to form a Cx-HA depot.

**Figure 2:**
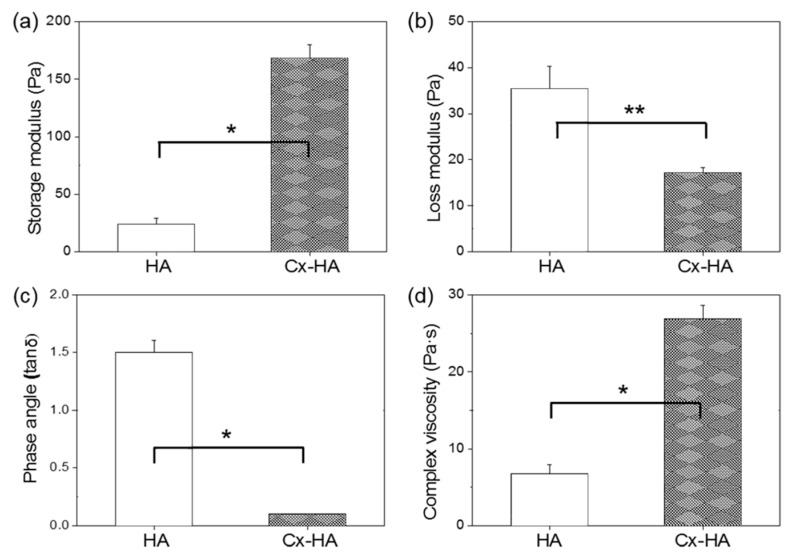
Rheological properties of HA and Cx-HA hydrogels. (**a**) Storage moduli, (**b**) loss moduli, (**c**) Phase angle (tan δ) and (**d**) complex viscosity (* *p* < 0.001, ** *p* < 0.01).

The rheological properties of Cx-HA and HA were monitored as the frequency was increased from 0.1 to 10 Hz (Figure 2). The storage (*G*′) and low loss (*G*″) moduli of HA and Cx-HA differed. The calculated phase angle (tan *δ*) based on the *G*″/*G*′ ratio of HA was greater than 1. In contrast, the tan δ values of Cx-HA were below 1, which indicated it possessed gel-like properties. The small phase angles indicated that the Cx-HA hydrogels were stronger than the HA hydrogels. The viscosity of Cx-HA was 4 times higher than that of HA. Taken together, the results indicated that Cx-HA was significantly stiffer and behaved more like a hydrogel than HA.

### 3.2. Confirmation of Cx-HA Depot Formation during Subcutaneous Injection

**Figure 3:**
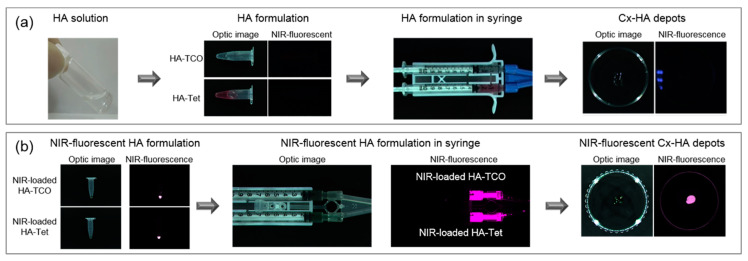
Optical and NIR fluorescence images of the injectable click-crosslinking HA formulation. (**a**) Optical and NIR fluorescence images of HA-TET and HA-TCO in tubes, a dual-barrel syringe and the Cx-HA depot obtained by injecting the formulation. (**b**) Optical and NIR fluorescence images of injectable click-crosslinking NIR-fluorescent HA formulation (HA-TET and HA-TCO with NIR-fluorescent) and the Cx-HA depot with NIR-fluorescent obtained by injecting the formulation.

To assess the injectability of the click-crosslinking HA (HA-TET and HA-TCO) formulation, HA-TET and HA-TCO were loaded into the separate compartments of a dual-barrel syringe and extruded through a 26 G needle (Figure 3a). No clogging in the extension tube was observed throughout extrusion of the HA-TET and HA-TCO solutions at a rate of approximately 4 μL/s. As HA-TET and HA-TCO were extruded from the dual-barrel syringe, they rapidly formed Cx-HA. The click-crosslinking HA (HA-TET and HA-TCO) formulation was thus injectable, and Cx-HA depot formation was reproducible. The facile preparation of the injectable click-crosslinking HA (HA-TET and HA-TCO) formulation was advantageous, and reproducible Cx-HA depot formation via the click-crosslinking reaction between TET and TCO occurred within seconds.

The HA-TCO solution was colorless, but the HA-TET solution had a slightly violet hue due to the natural color of TET. The Cx-HA depot did not fluoresce, which indicated that an NIR fluorophore would be needed for fluorescence imaging. The hydrophilic NIR fluorophore ZW800-1C dissolved readily in the HA-TET and HA-TCO solutions. After adding the NIR fluorophore to the HA-TET and HA-TCO solutions, the injectable click-crosslinking NIR-fluorescent HA (HA-TET and HA-TCO with NIR fluorophore) formulation was prepared by loading them into the separate compartments of a dual-barrel syringe. HA-TET and HA-TCO prepared with ZW800-1C both exhibited NIR fluorescence (Figure 3b). As the injectable click-crosslinking NIR-fluorescent HA formulation was extruded, an NIR-fluorescent Cx-HA depot immediately formed. The detection of fluorescence indicated that NIR fluorescence imaging of the Cx-HA depots would be possible.

**Figure 4:**
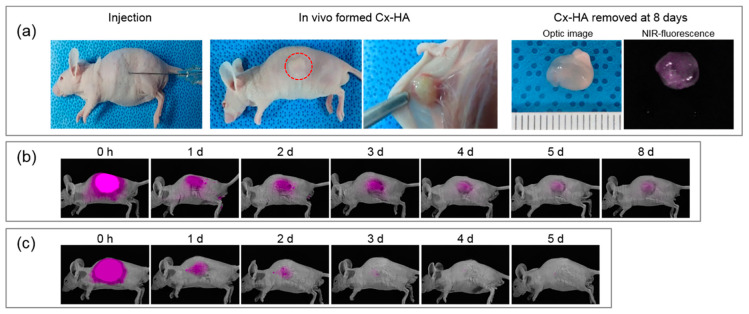
(**a**) Images of a mouse injected with the click-crosslinking NIR-fluorescent HA formulation and the resulting Cx-HA depot at 8 days. The Cx-HA depot was removed for optical and fluorescence imaging eight days after subcutaneous injection. NIR fluorescence images of mice injected with (**b**) the NIR-fluorescent HA formulation and (**c**) a solution of the NIR fluorophore. The images were acquired over a period of eight days following subcutaneous injection.

The click-crosslinking NIR-fluorescent HA formulation and NIR fluorophore solution was subcutaneously injected into mice, and the in vivo persistence of the Cx-HA depots was assessed by performing live NIR fluorescence imaging (Figure 4). NIR-fluorescent Cx-HA depots formed immediately at the injection sites.

In vivo fluorescence imaging was performed in real time to detect the Cx-HA depots and the injected NIR fluorophore solution. Fluorescence was observed for one day following injection of the NIR fluorophore solution, but no fluorescence was detected on day 4. In contrast, intense NIR fluorescence in the Cx-HA depots was immediately observed. The NIR fluorescence intensity in the images remained for the next eight days. These results indicated that the NIR-fluorescent Cx-HA depots were stable, and they persisted in vivo far longer than the NIR fluorophore solution.

The NIR-fluorescent Cx-HA depots were surgically removed from the mice eight days after injection to investigate the biocompatibility of the HA formulation. The NIR-fluorescent Cx-HA depots retained their original sizes. Blood vessels had formed on the Cx-HA depots, which indicated that they were biocompatible.

### 3.3. Confirmation of Cx-HA depot Formation during Intratympanic Injection

The click-crosslinking NIR-fluorescent HA formulation was loaded into a dual-barrel syringe for intratympanic injection, and a solution of the NIR fluorophore in saline was prepared to compare retention times. A syringe was used to create air vents in the tympanic membranes, and the animals were injected with either the click-crosslinking NIR-fluorescent HA formulation or the NIR fluorophore solution on the side opposite of the air vent. The intratympanic injection procedure was tolerated well by all of the rats. 

To investigate changes in the auditory brainstem response (ABR) during intratympanic injection of the click-crosslinking NIR-fluorescent HA formulation and the NIR fluorophore solution, the ABR of each animal was measured during stimulation of the injected ear and the opposite ear. Differences between the ABR values were then compared. The ABRs were stable, indicating that they were not significantly affected by injection of either the click-crosslinking NIR-fluorescent HA formulation or the NIR fluorophore solution.

**Figure 5:**
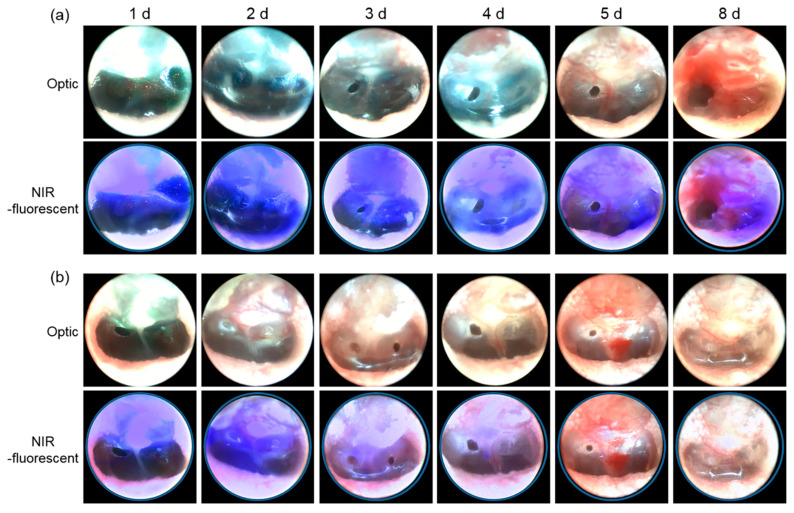
Optical and fluorescence images acquired after the intratympanic injection of (**a**) the click-crosslinking NIR-fluorescent HA formulation and (**b**) a solution of the NIR fluorophore.

NIR fluorescence imaging was performed following intratympanic injection (Figure 5). Fluorescence from both the click-crosslinking NIR-fluorescent HA formulation and the NIR fluorophore solution was observed immediately after intratympanic injection. 

The injected NIR fluorophore solution was clearly visible on day 1 and day 2, but it had begun to diffuse by day 3. No fluorescence was detected after day 5. Similar changes were observed in mice, in which no fluorescence from the subcutaneously injected NIR fluorophore solution was detected after four days.

In contrast, NIR fluorescence from the Cx-HA depots in the tympanic cavities was detected continuously for eight days. This confirmed that the injected click-crosslinking NIR-fluorescent HA formulation formed Cx-HA depots in the tympanic cavities, and that the NIR-fluorescent Cx-HA depots could be successfully imaged. This demonstrated that NIR imaging could be used to monitor Cx-HA depots in tympanic cavities for an extended period of time following intratympanic injection, although it is necessary to examine potential side effects such as degradation of Cx-HA depots in the tympanic cavities and patient convenience following intratympanic injection.

## 4. Conclusions

We successfully prepared an injectable click-crosslinking HA formulation and combined it with the NIR fluorophore ZW800-1C. The formulation can be easily prepared using a dual-barrel syringe, and it can be mixed with an imaging agent or potentially loaded with a drug. Click-crosslinking led to the rapid formation of depots at the tympanic cavity injection sites. The residence times of the depots were investigated by acquiring NIR fluorescence images of the tympanic cavity injection sites over time. Our results suggest that our injectable click-crosslinking HA formulation is a promising candidate as a depot for intratympanic drug delivery. Biodegradation and potential side effects of the intratympanic injected hydrogel in vivo and evaluation of treatment efficiency using steroids will be performed as future studies.
